# A “good death”: perspectives of Muslim patients and health care providers

**DOI:** 10.4103/0256-4947.62836

**Published:** 2010

**Authors:** Mohamad A. Tayeb, Ersan Al-Zamel, Muhammed M. Fareed, Hesham A. Abouellail

**Affiliations:** From the North West Armed Forces Hospitals, Tabuk, Saudi Arabia

## Abstract

**BACKGROUND AND OBJECTIVES:**

Twelve “good death” principles have been identified that apply to Westerners. This study aimed to review the TFHCOP good death perception to determine its validity for Muslim patients and health care providers, and to identify and describe other components of the Muslim good death perspective.

**SUBJECTS AND METHODS:**

Participants included 284 Muslims of both genders with different nationalities and careers. We used a 12-question questionnaire based on the 12 principles of the TFHCOP good death definition, followed by face-to-face interviews. We used descriptive statistics to analyze questionnaire responses. However, for new themes, we used a grounded theory approach with a “constant comparisons” method.

**RESULT:**

On average, each participant agreed on eight principles of the questionnaire. Dignity, privacy, spiritual and emotional support, access to hospice care, ability to issue advance directives, and to have time to say goodbye were the top priorities. Participants identified three main domains. The first domain was related to faith and belief. The second domain included some principles related to self-esteem and person>s image to friends and family. The third domain was related to satisfaction about family security after the death of the patient. Professional role distinctions were more pronounced than were gender or nationality differences.

**CONCLUSION:**

Several aspects of «good death,» as perceived by Western communities, are not recognized as being important by many Muslim patients and health care providers. Furthermore, our study introduced three novel components of good death in Muslim society.

The basic duty of health care providers is to preserve human health and life, but we should realize that death is the inevitable destiny of mankind predetermined by Allah (the creator of the universe). In cases in which recovery is hopeless, the role of health care providers does not end but rather is modified.^1^ Health care providers need to help patients and families minimize suffering and maximize comfort by offering appropriate medical care that is neither excessive nor negligent. This principle cannot be applied in 10% to 20% of cases in which death happens suddenly (myocardial infarction, arrhythmia, intracranial bleeding, aortic dissection, accident), but in 80% to 90% of cases, in which death is predicted,^2^ both the patient and the medical team are aware of its imminence. This is known as anticipated death, which must be planned if all goes well.

The whole subject of death and dying has been addressed in the West by pioneers such as Elisabeth Kubler-Ross and Raymond Moody: “Looking deeply into the way that we care for the dying person, they have shown that with unconditional love, and a more enlightened attitude, death can be a peaceful, even transformative experience.”^3^ However, dealing with a patient at the end of life, aiming to improve quality of life, and planning for end-of-life care requires a good understanding of that patient's state from a medical, social, spiritual, and psychological point of view. To date, this important topic is understudied in schools of medicine, and the extensive literature on medical errors shows that medical experts are rarely blamed for “bad” deaths.^4^ The authors of the final report on The Future of Health and Care of Older People (TFHCOP) have identified 12 principles of a good death (Table 1).^5^,^6^

**Table 1:** Principles of a end-of-life care according to the report, *The Future of Health and Care of Older People.*

To know when death is coming, and to understand what can be expected. To be able to retain control of what happens. To be afforded dignity and privacy. To have control over pain relief and other symptom control. To have choice and control over where death occurs (at home or elsewhere). To have access to information and expertise of whatever kind is necessary. To have access to any spiritual or emotional support required. To have access to hospice care in any location, not only in hospital. To have control over who is present at the time when the end comes. To be able to issue advance directives, which ensure wishes are respected. To have time to say goodbye, and control over other aspects of timing. To be able to leave when it is time to go, and not to have life prolonged pointlessly.

Most of the conducted studies examined the concept of good death in elderly whites, but no comparable qualitative data exist regarding other races.^7^ No qualitative study has been conducted in a Muslim society, and published papers on this important topic have focused mainly on reviewing Islamic teachings and the literature.^8^

The purpose of this study was to review the TFHCOP perception of good death to determine its validity for Muslim patients and health care providers and to identify and describe other elements for a good death. We tried to elaborate on the concept of good death in our society. The Western definition of “good death” was chosen rather than the Ghanaian or Tibetan one since our medical practice depends mainly on Western references; however, there should be some degree of flexibility in applying the Western perspective since death beliefs have religious and cultural backgrounds.

## SUBJECTS AND METHODS

Participants were recruited by a random sampling technique per category from King Abdul-Aziz Military Hospital and from a rehabilitation center and long-stay nursing unit in King Khalid Military Hospital in Tabuk. Since most people die surrounded by medical professionals, even when dying occurs at home, a full spectrum of persons involved with end-of-life care-physicians, nurses, social workers, religious officers, patients with life-limiting diseases, and their home caregivers (family members) were included in the survey.^12^,^13^ Patients and family members were recruited mainly from the Hematology and Oncology Center, especially those with advanced malignancy or those who were under palliative care. Death is a multi-dimensional process that involves physical symptom management as well as certain religious, social, and psychological needs of the dying and their loved ones.^14^ Non-physical issues may differ widely, which can be attributed mainly to religious and cultural differences. Therefore, we expected that Muslim participants may perceive these issues in a completely different manner. Approval of the study was obtained from the local research ethics committee.

Each participant was interviewed as well as administered a questionnaire. Before they were administered the questionnaire, they were made privy to the nature of the research and its purpose by asking the following questions: 1) What constitutes a good death and a bad one? 2) Can we develop our own definitions in keeping with our religious principles and cultural values? 3) Is this 12-principle definition valid as a definition of a good death in Islamic culture? 4) Please express your opinions freely. Do not be influenced by others' opinions. 5) Share your own experiences and feelings, and not what you have heard from others. 6) Express your opinions. There is no wrong or right answer. 7) We would like to know your opinions.

We developed a questionnaire on the basis of the TFHCOP's “12 principles of a good death.” The questionnaire comprised 12 closed-ended questions to which the participants had to answer in “Yes” or “No.” The questionnaires were provided in Arabic or English, depending on the participants' preferences (Appendices 1, 2). To ensure the validity of the questionnaire, before conducting the main interviews, we analyzed the questionnaire in both languages with 10 participants of different subgroups. We ensured that the questions were simple and easy to understand, so that the respondents did not have any difficulty in interpreting and answering the questions. Some participants, however, had difficulty in choosing a reply to a few questions. In such cases, we clarified the questions and explained each option by giving examples. We then let them determine how essential they found the point and to choose their answer accordingly.

**Appendix 1:**
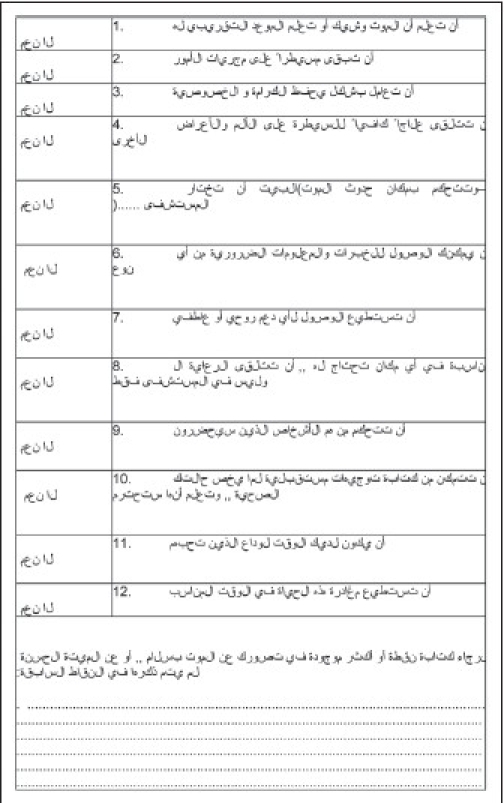
Questionnaire in Arabic.

**Appendix 2:**
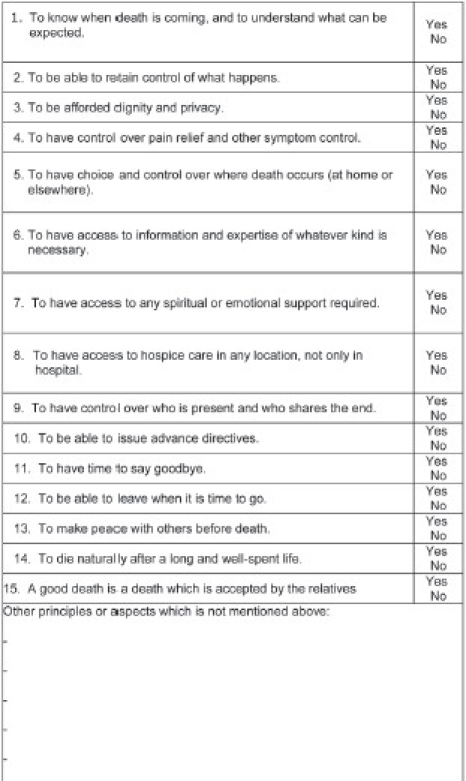
Questionnaire in English: What do you think about these principles? Do you think they are well-matched to your conception of a “good death?”

We used a qualitative approach to draw out previously unexplored aspects and components of the Muslim perspective. We conducted in-depth, open-ended, and face-to-face interviews and content analysis. We did not impose any theoretical assumptions a priori and instead let the participants suggest any principles that they considered to be essential.

After completing the questionnaire, the participants were asked to express and write their opinions on the principles (not included in the previous 12 points) that constitute a good death. We convened focus groups, each comprising two to seven participants, over a 2-month period. The interviews usually lasted for around 30 minutes per group.

Univariate descriptive statistics (frequencies, percentages) were used to analyze responses to each point of the questionnaire. Percentages are reported as whole numbers. For bivariate analysis, differences in participant characteristics among different questions were determined by using the chi-square test. *P* values of <.05 were considered statistically significant. For new themes we followed a grounded theory approach with a “constant comparisons” method and its related open and axial coding techniques.^9^,^10^ Trustworthiness was noted when participants respond affirmatively to researchers' interpretations.^11^

## RESULTS

Researchers interviewed 284 participants. Focus group participants ranged in age from 18 to 71 years (mean age, 37 years). The study sample consisted of 164 males (58%) and 120 females (42%). The study included participants of several different nationalities: 147 Saudi (52%), 42 Egyptian (15%), 23 Syrian (8%), 17 Pakistani (6%), 12 Sudanese (4%), 11 Filipino (4%), 11 Jordanian (4%), 10 Malaysian (4%), 7 Indian (3%), 2 South African (1%), 1 Moroccan (<1%), and 1 Nigerian (<1%). The most common groups among the participants were 93 nurses (33%), 88 physicians (31%), 43 patients' relatives (15%), 26 patients (9%), 16 social workers (6%), 14 clinical pharmacists (5%), and 4 hospital religious officers (1%).

Some focus group members were concerned about our society's tendency to consider death as taboo, something that human beings cannot interfere with. Participants informed us of cases in which health care providers avoided end-of-life discussions because they believed that it is beyond our control as humans.

On average, each participant agreed with the eight principles of the questionnaire, so we selected the top eight as the important principles for our society. Table 2 shows the frequencies and percentage of “yes” answers for each principle according to nationality, gender, and career. Health care providers were categorized into two groups: Group 1 included health care providers who are involved mainly in medical issues, i.e. physicians and nurses; Group 2 included the other members of the multi-disciplinary care team who are involved mainly in other aspects of care, i.e. patients' relatives, social workers, clinical pharmacists, and hospital religious officers.

**Table 2:** Number and frequencies of responses to each principle by group.

	Total	Sex		Nationality		Career		Patients
		Males	Females	Saudi	Non-Saudi	Health care providers Group 1	Health care providers Group 2	
	284	164	120	147	137	181	77	26
Principle 1: Timing of death	80 (28%)	48 (29%)	32 (27%)	41 (28%)	39 (28%)	50 (28%)	24 (31%)	26 (23%)
Principle 2: Control of what happens	177 (62%)	99 (60%)	78 (65%)	90 (61%)	87 (64%)	115 (64%)	47 (61%)	16 (62%)
Principle 3: Dignity and privacy	271 (95%)	156 (95%)	115 (96%)	144 (98%)	127 (93%)	176 (97%)	71 (92%)	24 (92%)
Principle 4: Pain and other symptom relief	246 (87%)	141 (86%)	105 (88%)	132 (90%)	114 (83%)	163 (90%)	60 (78%)	23 (88%)
Principle 5: Where death occurs	127 (45%)	70 (43%)	57 (48%)	70 (47%)	57 (41%)	86 (48%)	30 (39%)	11 (42%)
Principle 6: Access to necessary information	116 (41%)	50 (30%)*	66 (55%)*	63 (43%)	53 (39%)	82 (45%)*	21 (27%)*	13 (50%)*
Principle 7: Spiritual or emotional support	255 (90%)	146 (89%)	109 (91%)	135 (92%)	120 (88%)	166 (92%)	66 (86%)	23 (88%)
Principle 8: Access to hospice care	255 (90%)	144 (88%)	111 (93%)	135 (92%)	120 (88%)	165 (91%)	66 (86%)	24 (92%)
Principle 9: Control over who is present	108 (38%)	64 (39%)	44 (37%)	59 (40%)	49 (36%)	71 (39%)	30 (39%)	7 (27%)
Principle 10: To issue advance directives	240 (85%)	139 (85%)	101 (84%)	126 (40%)	114 (83%)	155 (86%)	63 (82%)	22 (85%)
Principle 11: To say goodbye	209 (74%)	119 (73%)	90 (75%)	108 (73%)	99 (72%)	133 (73%)	57 (74%)	19 (73%)
Principle 12: To leave when it is time to go	207 (73%)	110 (75%)	97 (71%)	109 (74%)	98 (72%)	137 (76%)	50 (65%)	20 (77%)

Participants identified certain aspects of quality of death that are not mentioned in the Western literature but prove to be essential for Muslims. These can be summarized in three main domains:

### Religious faith and beliefs

The first domain is related to a Muslim's faith, belief, and preferences during the dying process. This includes matters like 1) being sure that somebody is there to prompt him with *Shahadah* (bearing witness that there is no true God but Allah and Muhammad is verily His Servant and His Messenger) as a final statement of faith, 2) the presence of someone at the bedside to recite chapters of the Noble Qur'an, 3) to die in a position facing the holy mosque in Makkah, 4) to die in a holy place (e.g., Madinah, Makkah, or mosque) or in a holy time (e.g., in Ramadan or on a Friday).

### Self-esteem and body image

The second domain includes some principles related to the patient's self-esteem and image in his friends' and relatives' eyes, by avoiding post-mortem distortions, deformities, septic wounds, or bad odors by maintaining continence and keeping the body and clothes free of urine, stool, and vomit, and making sure the body has a normal appearance after death.

### Concerns about family security

The third domain is related to the patient's satisfaction about his relatives. In other words, he needs to feel that his family will be secure and have no trouble after his death, so he will not be worried about them. This relates primarily to economic and social concerns.

**Figure 1:**
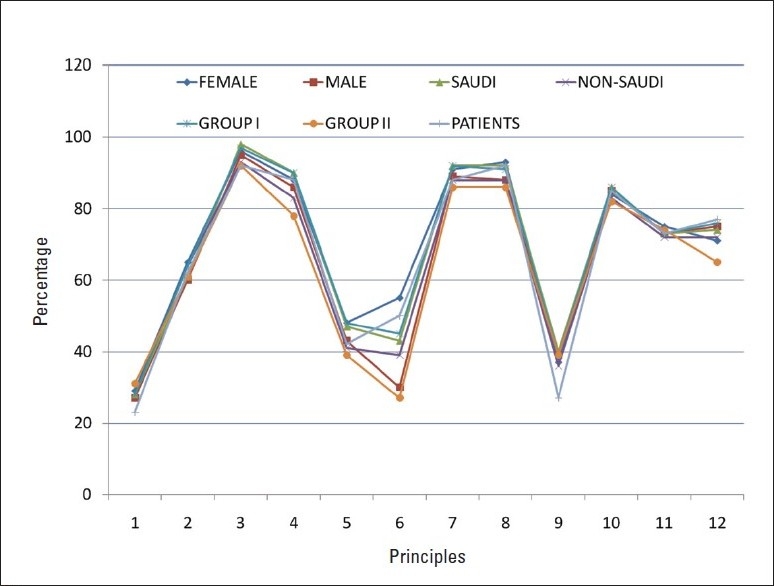
Percentage of responses to each principle by group.

It is worth mentioning that data obtained from a sub-group of Saudis reflected the same eight principles selected by major subgroups including females, health care professionals (Group 1), non-medical health care providers (Group 2), patients' home caregivers, and patients themselves (Figure 1). However, the order of sequence of these selected eight points was slightly different. The resultant responses were almost identical to all questions with minimal differences among groups. On the other hand, the three new themes reflect the common ground shared by participants. However, we found remarkable differences among groups. In fact, professional role distinctions were more pronounced than were gender or nationality differences. For example, most physicians and nurses were highly attuned to the needs of good hygiene and preservation of a patient up until his last breath. Religious officers discussed faith and spiritual support and were the only group to discuss postmortem preservation of configuration. Social workers' discussions were more psychosocial in nature and focused on being reassured about the family's future after their death. All themes were present in the patient focus group, but this group showed more concern about faith and their relationship with Allah Almighty.

## DISCUSSION

This study reveals that several aspects of “good death” as perceived by Western communities are not recognized as being of special importance by many Muslim patients and health care providers. Furthermore, our study introduced three novel components of good death in Muslim society. The definition of “good death” that is acceptable to Muslim patients and health care providers consists of 11 principles (Table 3). There was some kind of consensus on the importance of dignity and privacy, which can be explained by the Islamic perspective that respects human dignity and privacy and regards each as a fundamental pillar of Shari'a (Islamic Law). Secondly, Muslims by and large value spiritual and emotional support. They also believe that death is closely linked to faith. Therefore, most participants appreciated the importance of access to any needed spiritual or emotional support.

**Table 3:** Principles of a good death from the Muslim perspective.

Aspects related to faith and relationship with Allah Aspects related to self-esteem and person's image in the eyes of relatives Aspects related to concerns about family security To be afforded dignity and privacy To have access to any spiritual or emotional support required To have access to hospice care in any location, not only in hospital To be able to have control over pain relief and other symptom control To be able to issue advance directives, which ensure wishes are respected To have time to say goodbye and control over other aspects of timing To be able to leave when it is time to go, and not to have life prolonged pointlessly To be able to retain control of what happens

Control over pain and other symptoms is generally required by most people.^14^ Muslims perceive suffering as atonement for one's sins. This interpretation helps patients and family members cope with diseases. However, it does not belittle the fact that every effort should be made to relieve suffering. In our study, we met participants who prefer not to receive pain management because they believe that if a patient feels pain and shows patience, he will be rewarded more by Allah and will be more pure. We have to respect this wish because patients are granted the full right to accept or to refuse medical intervention despite the fact that Islamism encourages seeking treatment and there is a lack of religious evidence to support this point of view.^8^ Principle 4 may be modified to suit Muslim patients: “To have control over pain relief and other symptom control according to patient desire or preference.”

Further, while conducting interviews, we realized that some medical staff, including physicians, are not aware of “advance directives.” However, most of the participants preferred to issue advance directives, i.e. the right to refuse any therapeutic intervention, after the concept was explained to them. This study emphasizes that advance directives are underused in the medical practice of this hospital and that we have to encourage adopting it in our hospitals, especially since this study revealed that it is widely accepted by Muslim participants. It is very common in Arabic and Islamic societies to contact relatives and loved ones who may want to see the patient before death, but a physician has to suggest this when he feels that death is imminent and the patient is about to take his last breath. This is also standard practice in the West: staff members invite relatives to be present when death is impending to quietly say their goodbyes.^15^ If the number of visitors exceeds the available space, health care providers are expected to express a high degree of sensitivity if it becomes necessary to intervene in such situations.^8^

One cause of increased suffering is facing a lonely death in hospitals surrounded by strangers and advanced technologies and procedures that needlessly prolong the dying process without realizing the fact that the end of life is approaching.^16^,^17^ This may be a good reason why most participants chose to be able to leave when it is time to die and not to have life prolonged pointlessly. More importantly, Muslims do not generally believe that life is pointless, even when it is associated with a significant amount of suffering. They believe that Allah has the ultimate wisdom and is the most merciful and He will reward those who express patience and satisfaction when inflicted with disease or suffering. This, on the other hand, emphasizes the importance of being honest with patients, telling them about their prognosis, and giving them detailed explanations of Do Not Resuscitate orders. This may help in reassuring patients and makes them feel more comfortable that they will not receive any futile intervention.

Muslims believe that it is impossible to tell the time or place of death by any means and that it is only known by Allah Almighty who predetermines the exact timing and place of death. This may explain why the agreement rate for these principles was the least in this study, although many participants confirmed that it would be nice to have a “death alarm” that would enable the person to repent for his sins and ask for forgiveness. This supports the view that the patient and his family prefer to be given a less definitive answer. Surprisingly, if given a choice of a specific place to die, some participants repeatedly stated that they preferred to die in a holy place like a mosque, or in Makkah or Madinah. In this case, health care providers have to respect the patient's wish and may sometimes even be asked by the patient to help in persuading his/her family to grant this wish. This may be achieved by building a good rapport with the family and being honest and open with them regarding the patient's status, prognosis, and goals of care at that stage. This approach may help to decrease the patient's stress and prevent family members from feeling guilty about leaving him to die outside the hospital. Readers may note that we talk about the patient and his family as one unit, which contradicts Western practice and the concept of medical confidentiality. This could be attributed to the distinct nature of the Muslim family and its composite interrelations and strong ties.

This study has some limitations related to method and study design. A “Yes/No” question format may not be the best way to measure participants' preferences in this type of research. A five-point Likert scale would have been a better assessment tool because it would allow the participants to choose from a range of options that reflect the degree to which they agree or disagree with a statement and also to abstain from committing themselves to one response or the other if they were not sure. Although the fact that we obtained similar responses from all subgroups may support test reliability, further studies are required to assess the validity of using a questionnaire for this kind of topic. The questionnaire was provided in one of two languages, which may bear the potential for inter-rater variability and language translation inaccuracy. Researchers conducted a pilot study with 10 participants who received the questionnaire in one language and then in the other language. Proper modifications were made to ensure receipt of the same response. There was a preliminary assumption that principles of good death among whites, as reported by a TFHCOP, are applicable to a group of Muslim participants. This was obvious by adopting these principles as leading questions addressed to Muslim participants. The authors suggest that similar studies can be repeated using modified principles that are in better concordance with Islamic culture. Although our participants represented a broad range of ages, nationalities, careers, and educational levels, all were recruited from Northwest Armed Forces Hospitals; therefore, our findings may not reflect the perceptions in other areas. A large study on a national or international basis may be of importance to address this issue.

Our study has implications for both education and practice. Health care providers must understand their patients' preferences and respect their needs regardless of what the health care provider believes. Physicians and nurses should do their best to care for each patient's appearance and hygiene, as this is of great importance to Muslims. This can be achieved by keeping the clothes and body of the patient free from urine, stool, vomit, or blood whenever possible. They should be ready to help the patient to take a bath more frequently as well. To reduce postmortem disfigurement, jaw fixation and eye closing must be done immediately at the time of death. All patients should be reassured that this is the practice with all deceased Muslims in the hospital. The rites of washing, shrouding, funeral prayers, and burial should follow as soon as possible. In addition to expressing empathy, the major role of the health care team at this stage is timely documentation to prevent any unnecessary delay in proceeding with funeral rites. Physicians should be reminded that they are not alone when caring for dying patients; many other health care providers (nurses, social workers, and religious officers) are available to provide comprehensive care. Physicians may ask a screening question regarding any faith-related concerns and then ask whether the patient or relatives would like to speak in greater depth with a religious officer (a Sheikh or Imam). In addition, the physician should ask the patient a screening question to figure out any concerns related to his relatives and make sure that he has no other worries. The physician can then consult the social worker to help him in this respect. However, the palliative care team may need to identify families with suboptimal resources, provide the necessary support during bereavement, and reassure the patient that his family will receive economic support after his death. This is not the physician's primary duty, yet he should explore this point and ask for proper consultation, if necessary.

It is important that the medical curriculum covers this issue in detail. The Islamic perspective of “good death” must be included in health care services, professional codes, and care plans or missions for end-of-life care organizations and institutions in Islamic countries. This is more complicated than simply translating Western literature and trying to apply it to our practice that exists in a society with different cultural and religious backgrounds; care must be taken to develop such information in a Muslim-centered manner.
